# Toward a Differentiated Conceptualization of Extreme Experiences and Their Relevance To Integrative Mental Health Theory

**DOI:** 10.1007/s12124-025-09931-6

**Published:** 2025-10-30

**Authors:** Dominik Stefan Mihalits

**Affiliations:** 1 Faculty of Psychotherapy Science, Sigmund Freud University, Vienna, Austria; 2 Faculty of Psychology, Sigmund Freud University, Vienna, Austria; 3 Faculty of Humanities and Social Sciences, Aalborg University, Aalborg, Denmark

**Keywords:** Extremes in science, Extreme term definition, Modularity of extremes, Cultural narrative of extreme, Subjectivity within extremes, Diagnostic expansion, Clinical implications of extremes

## Abstract

Extreme experiences lie at the edges of human existence—and precisely for that reason, they demand the attention of psychology and psychotherapy science. However, in this context, extremes are often interpreted as pathological deviations from an assumed norm. This article argues for a conceptual re-evaluation: extremes should not be understood primarily as symptomatic distortion or disorders, but as potentially meaningful states that are relevant to psychological development. Building on historical, theoretical, and empirical perspectives, an integrative framework model is proposed that analyzes extremes as multidimensional constructs: First, the dimension of *intensity* describes the extent of emotional, cognitive or physical arousal—from *overwhelming* overstimulation to emotional *numbness*. Second, the *impact* dimension reflects the extent to which an experience can lead to *transformation* or serve to restore and maintain psychological *stability*. Third, the *demand* dimension refers to the mental or physical effort required by a situation, ranging from states of intense *exertion* to those characterized by *ease*. Fourth, the *control* dimension concerns the subjective experience of *gaining* or *losing* control. Fifth, the *novelty* dimension describes the degree of unfamiliarity of an experience within an individual or cultural context, ranging from states of *newness* to those characterized by *ritualism*. And sixth, the *identification* dimension highlights the degree of personal connection to an experience, ranging from strong *involvement* to *detached* observation. The proposed model allows for a differentiated view of extreme experiences beyond binary categories such as “normal” vs. “clinically significant.” It understands extremes as a fundamental psychological phenomenon with both destructive and transformative potential. The goal is to develop a theory of extremes that is relevant for both psychological research and psychotherapeutic practice.

## Introduction

We seldom arrive at truth except through extremes; we must first exhaust error—and often madness—before we can attain the radiant goal of peaceful wisdom.”^1^

Schiller (1786/1845, p. 342).

In his philosophical letters, Schiller deals with the connection between aesthetic culture, moral obligation, and the development of human character. He stresses that true beauty leads to freedom and moral understanding. In his understanding, as the selected quote shows, Schiller recognizes that *extremes* play an important role in the development of knowledge and thus in the understanding of human beings and formation of character. Extremes, at least according to the classical literature that followed Schiller, are therefore necessary for understanding human discourse. His observation, as I aim to show in this article, may have been made without further intention and may have been considered merely a statement at the time; however, it remains valid until today. Extremes still seem to occupy a special place in societies and their sciences.

This paper addresses the question of how extreme experiences can be described in a psychologically differentiated way and theoretically modelled without being prematurely pathologized. The aim is to develop a structuring model that integrates both clinical-therapeutic and cultural-psychological perspectives, making the relevance of extreme experiences for psychological development visible. Although the term “extreme” is increasingly used in scientific language—for example, in medical, psychological, or social science contexts—there is still no theoretically coherent model that systematically describes these experiences and makes them integrable into psychotherapeutic contexts. This desideratum forms the starting point of the present work.

### Normal or Pathological?—Basic Ambiguity of Extremes

In the scientific study of psychological processes, a field has emerged that goes beyond clinical application and also reflects on psychotherapeutic phenomena from a theoretical, empirical, and cultural perspective: psychotherapy science. It integrates subjective, biographical, and social perspectives and recognizes—as Erismann (2019) emphasizes—contradictions and ambivalences as constitutive elements of scientific knowledge. Within this framework, extreme experiences are often recorded by diagnostic systems such as the DSM-5 (American Psychiatric Association, 2022) or the OPD (Operationalized Psychodynamic Diagnosis, (Working Group on Operationalized Psychodynamic Diagnostics, 2023)),^2^—particularly along conflict axes or structural models. These systems tend to classify extremes as deviations from an assumed norm, often leading to premature pathologization.

To concretize the theoretical discussion, it is helpful to name various manifestations of extreme experiences by way of example. These include religious ecstasies, near-death experiences, psychotic episodes, spiritual crises, artistic breakthroughs, extreme athletic performances, ritual initiations, and experiences in contexts of displacement, existential threat, or severely burdened living conditions. What these phenomena share is a tendency to involve intense emotional engagement, loss of control, shifts in identity, or profound meaning-making—regardless of whether they are interpreted as pathological, transformative, or culturally embedded. This diversity of extreme experiences raises the question of how psychological science defines and evaluates such phenomena. In doing so, it frequently fails to reflect on how these norms arise and who defines them (Wakefield, 1992).

Psychology also tends to treat extreme phenomena as marginal phenomena rather than understanding them as an integral part of psychological processes. In many subfields—such as clinical psychology, personality psychology, and social psychology—extremes are often treated as statistical outliers or anomalies. For example, clinical psychology interprets intense emotional states as an expression of affective dysregulation (e.g., in the context of borderline personality disorder), while social psychology understands radical behaviors as deviations from group norms or as expressions of cognitive dissonance. These interpretations are also often based on a binary logic of normality versus disorder.

The tendency in psychology and psychotherapy science to interpret extreme phenomena as pathological deviations from a statistically, diagnostically, or culturally constructed norm is not only theoretically questionable but also methodologically problematic. Particularly relevant in this context is Paul Meehl’s (1967, 1978, 1990) critique of “soft psychology”: He showed that the widespread use of null hypothesis testing in these fields often leads to epistemically unreliable conclusions.

The “obfuscating” factors identified by Meehl (1990), which complicate the interpretation of research findings within the sciences of mind and behavior, are not merely methodological challenges—they are also highly relevant for understanding extreme phenomena. Research on psychological extremes in particular shows how easily such states can be prematurely classified as pathological if the theoretical and methodological foundations are insufficiently reflected upon. The theory of extremes developed here, is therefore also intended as a response to these methodological weaknesses—it offers a differentiated, context-sensitive model that systematically captures the complexity of extreme experiences. While not all of Meehl’s obfuscating factors are addressed in detail here, the theory of extremes engages selectively with those most relevant to the conceptual and methodological challenges surrounding extreme psychological phenomena.

According to Meehl, a central problem lies in the *loose derivation chain* between theory and hypothesis. In many studies, it remains unclear how exactly theoretical assumptions can be translated into empirical predictions. For extreme phenomena, this means that without a clear definition of what an “extreme state” is, any deviation from the norm is potentially pathologized. A structured theoretical framework, as sought in this work, can help to close such gaps in derivation and avoid prematurely classifying extreme phenomena as mere deviations, instead capturing them in a differentiated and context-sensitive manner.

Added to this are *problematic auxiliary theories*, for example on the validity of measuring instruments or the effect of experimental manipulations. For example, if a test for “emotional stability” is used without clarifying whether it can actually measure extreme emotional states, the result will be unreliable. The theory of extremes therefore requires explicit reflection on the methodological prerequisites and contextual validation of the instruments used, inclusive of the cases of crossing over beyond the borders of stability.

The *ceteris paribus clause*, i.e., the assumption that all other conditions remain constant, is particularly fragile in research on extremes. Extreme states often arise precisely from complex interactions between individual, social, and cultural factors. Moreover, many extreme experiences are not merely endured but actively sought out—through self-motivated acts such as spiritual quests, artistic immersion, or high-risk endeavors—making them inseparable from the agent’s intentionality and existential orientation. A model that systematically takes this context-dependence into account, as I propose, can counteract such simplifications. For example, religious ecstasy cannot be studied in isolation from an individual psychological perspective if it is deeply embedded in cultural rituals, spiritual attributions of meaning, and biographical crisis experiences that significantly shape the experience and become a prerequisite for such an experience.

*Experimenter errors*, such as unconscious influence by researchers or faulty implementation, can also distort results. When working with extreme phenomena, which are often accompanied by high emotional involvement, methodological rigor is particularly crucial—not least because the emotional involvement of the researchers themselves can also lead to distortions. The theory of extremes therefore advocates a reflexive research attitude that is aware of its own positioning and its potential impact on the research process.

The so-called *crud factor*—the observation that in psychology, almost everything correlates with everything else—means that even random correlations can appear significant. For extreme phenomena, this means that a correlation with a “diagnostic criterium” can be mistakenly interpreted as evidence of pathology. The dimension-based model of extremes helps to distinguish such spurious correlations from genuine psychological dynamics.

The *selective practice in pilot studies* further contributes to the bias: studies that do not yield significant results are often not pursued or published. This creates a one-sided picture in which extremes only become visible when they appear problematic. The theory of extremes, on the other hand, calls for openness to ambivalence and ambiguity—including in research practice.

This *bias* is reinforced by *selective paper submission*: researchers tend to submit only studies with positive results. This reinforces the tendency to focus on extremes only when they appear pathological or in need of treatment. The theory of extremes advocates normalizing the extreme—including in scientific presentations.

*Editorial publication bias* also plays a role: journals favor significant and often pathologizing results. This leads to a literature in which extremes appear almost exclusively as expressions of disorder. The theory developed here counters this one-sidedness with an integrative understanding that also makes positive, creative, or transformative extremes visible.

Finally, Meehl warns against the *uncritical acceptance of validity claims for psychometric instruments*. If a test is considered “valid” even though it inadequately reflects complex phenomena such as extremes, the findings derived from it can be misleading. The theory of extremes therefore calls for a differentiated, dimension-based operationalization that goes beyond one-dimensional scales and includes subjective and cultural aspects.

Overall, it is clear that the ten obfuscating factors described by Meehl are not just methodological pitfalls—they actively contribute to extreme phenomena being misunderstood, marginalized, or pathologized in psychology and psychotherapy.

### Extremes: Developments Beyond the Ordinary

The theory of extremes developed here is also intended as a methodological correction: it aims to understand extreme experiences not as statistical outliers, but as complex, context-bound, and potentially meaningful expressions of human development. The method used in this work consists of a theoretical conceptualization based on a systematic analysis and integration of relevant historical, theoretical, and empirical literature. The model developed in this work follows the approach of conceptual theory formation as outlined by Jaccard and Jacoby (2010). It constitutes a heuristic model that is not based on empirical content analysis, but rather on the systematic integration and theoretical abstraction of existing literature. Heuristic models serve as cognitive tools that help to structure complex phenomena, generate new hypotheses and open up new research perspectives. They are not intended as definitive explanations, but rather as structuring frameworks that deepen and differentiate the understanding of a given phenomenon—in this case, extreme experiences. In this sense, the model can also be understood as a cognitive framework, treating categories not as objectively given entities but as mental constructions that organize our understanding of the world (Lakoff, 1987). The six dimensions of the model represent semantic fields that allow for a differentiated grasp of extreme experiences and their integration into psychological and cultural contexts. This approach aligns with broader critical perspectives that question conventional definitions of normality and deviance.

Similarly, in his historical analysis of madness, Foucault (1988) shows how definitions of normality and madness are socially constructed, with “extremity” often being used as a mechanism of exclusion. In this light, extremes are not just a matter of measurement, but also products of institutional authority and cultural consensus. This has profound implications for how psychology and psychotherapy identify and respond to extreme behavior and requires a more critical and reflexive attitude toward what is considered deviant or disordered.

While the prevailing discourse tends to view extremes as dysfunctions to be corrected, alternative paradigms suggest that extreme states can be not only meaningful but even necessary for change. From a Jungian perspective, for example, the path to individuation—that is, to the development of an integrated self—is inextricably linked to the conscious confrontation of unconscious aspects of the personality (Jung, 1963). This confrontation rarely proceeds without conflict: it is often accompanied by profound tensions, symbolic moments of crisis, and encounters with the so-called shadow—those repressed aspects of the self that powerfully reassert themselves in extreme states.

According to Jung, the self is not a static state, but a goal that can only be achieved through the conscious working through of inner contradictions. Extreme experiences often mark threshold moments in this process, in which the ego questions its previous structures and has to realign itself. These liminal states—psychological as well as symbolic—can be understood as productive crises in which the psychological system reorganizes itself. Archetypal images, as they appear in dreams, myths, or religious symbols, function as psychological signposts (Jung, 1953). They help to interpret what has been experienced and integrate it into a new self-structure. Analytical psychology does not regard such images as mere cultural artifacts, but as expressions of universal psychological dynamics that are particularly evident in extreme experiences: “One does not become enlightened by imagining figures of light, but by making the darkness conscious (Jung, 1967, p. 335)”. Therefore, self-confrontation is not an exceptional state, but a constitutive part of human development. It often leads to extreme psychological states—which in turn can have a traumatic effect. But it is precisely in this tension between disintegration and reorientation that the transformative potential lies: extreme experiences are not only a risk, but also a resource. This idea is also found in contemporary models of post-traumatic growth, which assume that people who experience extreme stress or trauma can undergo significant positive psychological development—such as increased personal strength, a new sense of meaning, or a deeper ability to form relationships (Tedeschi & Calhoun, 2004). What initially appears to be a breakdown can, over time, become a catalyst for a breakthrough. However, beyond this response to trauma, extreme experiences can also develop their own dynamic of development, independently of crises or injuries. Self-confrontation—in the form of spiritual seeking, artistic devotion or existential reflection, for example—is part of normal life and can lead to maturation, insight and reorientation. The extreme is not only a consequence of collapse, but also an expression of new beginnings. New findings from psychedelic-assisted therapy further complicate traditional views. Psychedelic experiences are often characterized by extreme changes in perception, emotions, and the dissolution of self-boundaries. Under controlled conditions, however, these experiences have shown therapeutic benefits, including a significant reduction in depression and anxiety (Carhart-Harris et al., 2014). Instead of eliminating extreme states, this approach harnesses their power for integrative and healing purposes—and thus makes it clear that the extreme can be understood not only as a disorder, but also as an opportunity.

### The Conceptual Blinders of Polarization or Why Extremism Misses the Point

Extreme experiences are ubiquitous in psychotherapy practice and clinical psychology—and yet they are often distorted in scientific discourse. Confusion over terminology, narrow diagnostic criteria, and cultural assumptions mean that extreme states are often hastily interpreted as pathological deviations. As shown in the previous section, there is currently no theoretical framework that understands extreme experiences not only as exceptional phenomena, but as potentially significant expressions of psychological development.

Extremes are potentially socially sensitive phenomena. In an age characterized by social polarization, media sensationalism, and binary thinking, the term “extreme” is also often equated with danger, radicalism, or ideological aberration. This is particularly evident in its proximity to the term “extremism,” which in public discourse is almost exclusively associated with political or religious radicalization, violence, and threats to society.^3^ However, this semantic overlap obscures the actual focus of this article: it is not about extremism as an ideological stance, but about extreme psychological experiences—that is, subjective states characterized by extraordinary intensity, changes in the experience of control, emotional overwhelm, or existential borderline situations. These experiences are not primarily motivated by a goal or ideology, but by inner dynamics, biographical ruptures, transitions, or explorations that are deeply rooted in the individual’s psychological structure. Such experiences often occur in existential crises—such as spiritual upheavals, traumatic experiences, artistic breakthroughs, or profound emotional transformations. They are not only an expression of disintegration, but also of reorganization: they mark threshold states in which the old no longer holds and the new is not yet tangible. In this sense, extreme experiences are not marginal phenomena, but potentially central stages in the course of human development.

The theory of extremes developed here starts at precisely this point: It understands extreme experiences as psychologically significant phenomena that destabilize existing interpretive frameworks and can thereby trigger processes of identity formation, meaning-making, and psychological transformation. To do justice to this complexity, a theoretical model is needed that does not reduce extreme experiences to ideological attributions or diagnostic categories, but systematically captures their structural characteristics. The theory of extremes presented below is an attempt to do just that: it does not seek to normalize or pathologize, but rather to differentiate—and thus contribute to a deeper understanding of the psychological dynamics at the edges of experience.

## Extremes are Usual—But We Need an Adequate Theory

The term *extreme* has experienced a remarkable boom in scientific language in recent decades. We encounter it in a wide variety of disciplines—from medicine to climate research to treatment and psychology—and it is used in a variety of contexts: to describe physical limits, social deviations, or emotional states of emergency. This semantic breadth points to a conceptual appeal that has, however, hardly been systematically reflected upon.

It is precisely this discrepancy between the widespread use of the term and its theoretical vagueness that forms the starting point for the following chapter. Using various examples—including a quantitative analysis of scientific publications (e.g., in PubMed), but also observations from everyday and technical language—we first show how frequently and inconsistently the term *extreme* is used. It becomes clear that there is neither an interdisciplinary definition nor a consistent theoretical framework to guide the use of the term.

This observation gives rise to three central theses: First, the term *extreme* seems to be gaining increasing relevance in scientific communication. Second, its meaning remains largely contingent and context-dependent. And third—and this is the guiding assumption of this chapter—this semantic openness could conceal a previously unrecognized structure that connects various phenomena. The following sections explore this hypothesis and thus prepare the ground for the development of a theoretical model that makes extreme experiences comprehensible not only as a linguistic category but also as a structural phenomenon.

### Science Comes to Look at the Extremes—But Slowly

**Fig. 1:**
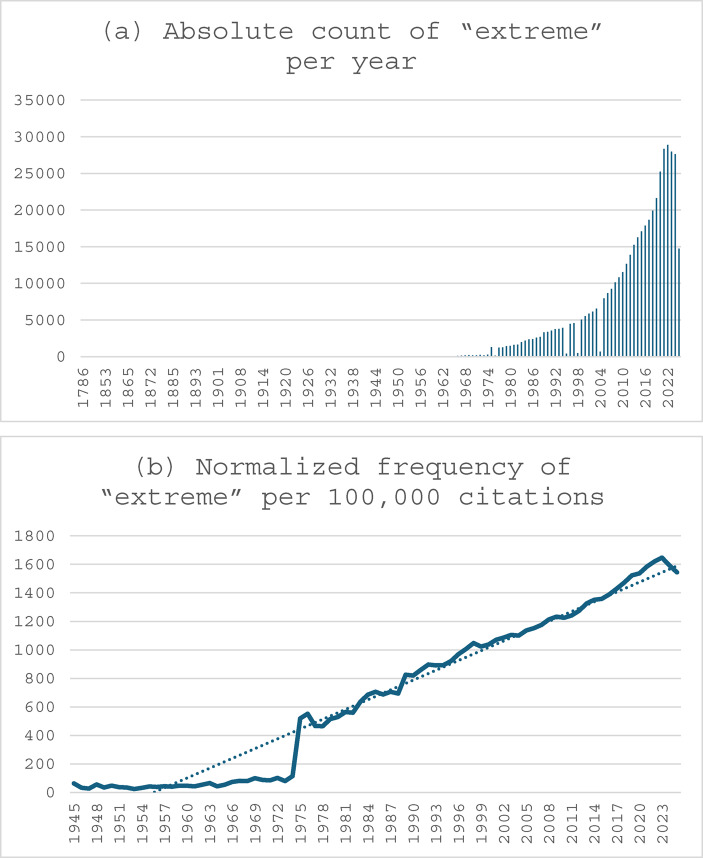
(a) Absolute count of PubMed citations containing the word “extreme” per year (N = 4,278,728; PubMed database N > 38 million). (b) Normalized frequency of “extreme” citations per 100,000 PubMed entries per year (proportion by year, 1945–2025). Source: PubMed search engine; query received 31/05/2024 18:11 CET. Retrieved from https://pubmed.ncbi.nlm.nih.gov on 30/06/2025 15:44 CET

Science is curious. Its primary goals is to depict and explain the world as precisely and accurately as possible. It follows that scientific publications strive to represent the world exactly as it is observed. This suggests that the role of extremes in scientific literature should be examined, as illustrated in the following examples. A simple search query in the scientific database PubMed illustrates how much the dissemination of materials dealing with extremes has increased over the last 150 years. The word “extreme” was used as the search term, which yielded a total of 4,278,728 hits. Specifically, this means that the word “extreme” appears in titles, subtitles, abstracts, or as a keyword in the publications. According to Fiorini, Lipman, & Lu (2017), PubMed indexed approximately 27 million articles at that time (currently 38 million articles at the time of the search), which initially makes the number of hits seem even less meaningful. Nevertheless, it seems remarkable that, relatively speaking, 11.26% of all texts in the history of PubMed use the word “extreme” in some form (see Figure 1a). However, looking at the absolute number in relation to the time of publication, it becomes clear that the use of the word “extreme” in research has increased significantly (not to say exponentially) over the last 50 years (since around 1974). The trend shown in Fig. 1b is particularly interesting in highlighting the relevance of the topic. Figure 1b confirms that this is not a random observation. It shows the number of hits for the word “extreme” normalized per 100,000 citations in the database. A clear trend can be seen, confirming the steadily increasing use of “extreme.” The following conclusions can be drawn from this study: The word “extreme” is enjoying growing popularity in scientific language. This example seems all the more striking when one considers that there are no clear definitions of extremes in everyday language.

The increasing prevalence of the term *extreme* in scientific language points to a growing interest in phenomena that lie at the margins of the ordinary—yet the term itself often remains theoretically underdefined. We encounter it in a wide variety of disciplines—from medicine to climate research to the human sciences—and it is used in a variety of contexts: to describe physical limits, social deviations, or emotional states of exception. However, this conceptual vagueness is not merely a shortcoming, but rather an indication of the complexity of the phenomenon: extreme experiences defy simple definitions because they are anchored not only in language, but above all in existence. They occur where familiar patterns of interpretation fail, where the self is shaken and new forms of orientation become necessary. In order to understand the psychological potential of such experiences, it is therefore not enough to analyze their linguistic use—it is necessary to turn to the lived experience itself. The following section is devoted to this perspective: the situational component of extreme experiences as they manifest themselves in concrete life contexts and can challenge or restructure the psychological (in-)balance.

## The Situational Component—or the Idea of the Extreme Situation

The human sciences deal with extremes in a rather one-sided manner, as the following example from the work of Bruno Bettelheim illustrates. Bettelheim, himself a psychologist and psychoanalyst, was imprisoned in the Buchenau concentration camp from May 1938 to April 1939 and is thus himself one of the victims of the Nazi regime. Thanks to the efforts of American supporters, he was able to emigrate to the US in 1939, where he pursued a university career and attempted to make sense of his experiences in his psychological work, but was highly controversial in doing so.^4^

The following section will therefore not examine or analyze the controversial findings of Bettelheim’s research, which already seem to have been sufficiently addressed, but rather the first-person narrative perspective that describes direct experiences and leads to an attempt at an initial definition, namely that of the extreme situation.

Bettelheim (1943, 1982) explains that an extreme situation arises when we suddenly find ourselves in a situation in which our previous coping strategies and familiar values fail—they can even become dangerous to us, even though they previously offered us protection. In such moments, we lose our familiar protective system and are forced to completely reorient ourselves. We have to develop new ways of thinking, new ways of life, and new values in line with the situation. He describes how he had exactly this experience—like many others in his story—in the spring of 1938, when he was arrested after German troops marched into Austria. In November of that year, tens of thousands of people suffered a similar fate when the Nazis organized a massive pogrom following the assassination of Ernst vom Rath.^5^

Bettelheim goes on to say that, in a way, he was better prepared for this shock than many of his fellow prisoners. His interest in politics had given him access to reports that had been smuggled out of the Third Reich and described life in the camps. Bettelheim vividly describes how certain experiences in concentration camps overwhelm the usual psychological coping mechanisms and necessitate new, often unconscious strategies. He writes:

*It seems that camp experiences which remained within the normal frame of reference of a prisoner’s life experience were dealt with by means of the normal psychological mechanisms. Once the experience transcended this frame of reference*,* the normal mechanisms seemed no longer able to deal adequately with it and new psychological mechanisms were needed.* (1943, p. 433)

This passage makes it clear that extreme experiences are defined not only by their intensity, but above all by their structural strangeness and transgression of boundaries. They break down the subject’s familiar frame of reference and require a psychological reorganization that goes beyond everyday coping strategies. In Bettelheim’s words, it becomes clear that the extreme lies not only in the extent of suffering, but in the qualitative shift in the experience of reality—a break with the familiar that forces new psychological mechanisms. This insight is central to the understanding developed here of extreme experiences as threshold phenomena that can not only destroy but also transform.

In addition, psychoanalysis had given Bettelheim a deeper understanding of the dark side of human nature—of hatred, destructive rage, and those powerful inner forces that Freud called the “death instinct”. A closer examination of Freud’s concept of the death drive^6^ (Freud, 1920) seems helpful here in order to better understand Bettelheim’s perspective. Freud describes the death drive as a fundamental, destructive force in humans that aims at dissolution, a return to the inorganic, and self-destruction. In extreme situations—such as concentration camps—this drive component not only becomes visible, but also structures people’s behavior. Bettelheim suggests that understanding these inner dynamics helped him to understand the behavior of fellow prisoners and perpetrators not only as morally reprehensible, but also as psychologically explainable. A more comprehensive presentation of the theory of death drive would require a thematic expansion that would shift the focus of this article too far away from the structural analysis of extreme experiences. However, the contextualization outlined here is sufficient to illustrate the relevance of psychoanalytic concepts for understanding inner psychological dynamics in extreme situations.

### Unconscious Dynamics and the Roots of Extremity

It is also worth taking a closer look at Freud’s reflections on narcissism (1914, 1917). Freud makes it clear at the outset that he is not talking about an intellectual difficulty, i.e., one that makes psychoanalysis difficult to understand. Rather, he is concerned with an emotional hurdle: Psychoanalysis is rejected by many because it hurts or irritates feelings, which leads to people paying less attention to it or trusting it less. Ultimately, both types of difficulty lead to the same result: those who are not emotionally open to something will find it more difficult to understand. Now, it is far from my intention in my work to promote psychoanalysis or to denigrate it. Rather, I am more concerned with sharpening our view of extremes and making the possibility of unconscious phenomena tangible as a potential possibility. This is because, in my opinion, it can provide possible explanations for the primary development of extremes. An understanding of narcissism seems to be a prerequisite for this. However, not from a psychopathological perspective, but rather from a developmental psychological necessity. Freud writes that psychoanalysis has developed a theory from many individual observations, which is generally known as libido theory. It deals with the study and treatment of so-called nervous disorders. In order to understand these, he turned to the dynamics of the drives as the foundation of psychic life. The assumptions about human drives thus form the basis for understanding nervousness. Freud postulated at the time that conventional scientific psychology provided only inadequate answers to questions about the inner life, especially when it came to drives. Freud generally distinguishes between drives that serve self-preservation (e.g., hunger) and those that aim at reproduction (e.g., love). Psychoanalysis adopts this distinction and speaks of ego drives and sexual drives. The energy of the sexual drive, called libido, is compared to expressions of hunger or the ego drives’ striving for power. On this basis, a central insight is gained: sexual drives are crucial to understanding neurotic disorders. Neuroses are essentially disorders of sexual function. Whether someone develops a neurosis depends on how much libido they have and whether it can be satisfied. The type of disorder, in turn, results from the individual development of sexual function—in particular from what are known as libido fixations. Freud believed that psychoanalysis had developed a special technique for recognizing and treating this type of conflict. It is therefore particularly successful in treating neuroses that arise from a conflict between ego and sexual drives. The ego often perceives sexual drives as a threat to its self-esteem or self-preservation and suppresses them. These drives then seek detours in the form of substitute gratifications, which manifest themselves as symptoms. Psychoanalytic therapy attempts to reverse this repression process and steer the inner conflict in a healthier direction. Critics accuse psychoanalysis of attaching too much importance to sexual drives. But this is a misunderstanding: psychoanalysis does not neglect other human interests—it merely focuses on one central aspect, just as a chemist concentrates on chemical forces without denying gravity and while still acknowledging physics. In therapy, the objects to which the libido is bound are analyzed in order to release them and make them available to the ego again. This gave rise to the image of an original distribution of libido: at the beginning, all libido is directed toward the self—a state that Freud calls primary narcissism. Only later, through experiences with the outside world, is the libido transferred to external objects. Secondary narcissism, on the other hand, describes a state in which an individual who has already established object relationships withdraws the libido from the object and redirects it back to their own ego. A central element of his theory is the distinction between ego libido and object libido. While ego libido is directed toward the self, object libido refers to other people or things. According to Freud, narcissism arises when ego libido predominates—that is, when libidinal energy remains directed inward or returns inward rather than being directed outward. The transition from narcissism to object love is therefore considered a developmental step. Nevertheless, part of the libido always remains with the ego—a certain degree of narcissism persists. The ego is a reservoir from which libido flows to objects and returns to it. It is crucial for mental health that this mobility is maintained. This theory—the libido theory of neuroses—forms the basis for understanding and treating mental illness. It also applies to normal behavior. For example, we speak of the narcissism of children or primitive humans, who believe in the omnipotence of their thoughts and want to influence the world through magic. The role played by extremes remains unclear. Freud goes on to explain that humanity has suffered three severe blows through science:

- Cosmological Blow^7^: Humans believe that the Earth is the center of the universe. This illusion was destroyed by Copernicus, who was able to show that the Earth revolves around the sun.
- Biological Blow^8^: Humans see themselves as higher beings above animals. However, Darwin was able to prove that humans evolved from animals and are similar to them in many ways.
- Psychological Blow^9^: Humans consider themselves masters of their own souls. But psychoanalysis shows that the ego does not rule over all mental processes. Thoughts and impulses arise that seem foreign to consciousness and elude the will. These symptoms are expressions of repressed drives that assert themselves unconsciously.

Psychoanalysis explains that these foreign thoughts are parts of one’s own soul life that have withdrawn from consciousness. The ego fights against itself, not against an external enemy. The cause lies in the overestimation of one’s own control over sexual drives. These have taken on a life of their own and manifest themselves in symptoms that the ego no longer recognizes as part of itself. Another mistake is to believe that everything important in the soul is communicated to the consciousness. But many processes remain unconscious. Consciousness is incomplete and often unreliable. We only learn many things after they have already happened. Freud demands: Know thyself, go deep within yourself—only then can you understand why you become ill and perhaps prevent yourself from becoming ill. Psychoanalysis aims to enlighten the ego. However, its two central statements—that sexual life cannot be completely controlled and that the soul is largely unconscious—mean that the ego is not master in its own house. This realization is the third narcissistic injury. The idea of unconscious mental processes is a profound turning point for science and life. Freud emphasizes that philosophers such as Schopenhauer had already anticipated this idea—for example, with his concept of the unconscious will. Psychoanalysis goes further, however: it proves these theses not only theoretically, but also with concrete cases.

### From Libido to Liminality: Psychoanalytic Insights into Extremes

Freud’s theoretical approach, in particular his conception of libido (Freud, 1905, 1916–1917) as a source of psychic energy, offers a fruitful starting point for understanding extreme experiences: While Freud initially understood libido as a drive energy directed toward sexual objects, his thinking evolved toward a more comprehensive view of inner conflicts that can manifest themselves in neurotic symptoms, but also in creative or spiritual forms of expression. In this context, it can be argued that extreme experiences—such as those that occur in religious ecstasy, ritual self-harm, or spiritual transgressions—can be understood not only as pathological phenomena, but also as expressions of a profound inner dynamic. When the libido, originally directed toward external objects, can withdraw in times of crisis and reorganize itself in the form of regressive or narcissistic states. But it is precisely in these moments of inner disorientation that transitional spaces open up, which I would like to describe below as liminal states in which new meanings, identities, and self-relationships can emerge. Such liminal spaces are thus no longer to be understood as merely situational and static or as passive, but prove to be an ongoing and progressive psychological process.

Based on Freud’s concepts and Bettelheim’s experiences, a transition can be identified: from passive confrontation with extreme situations to active self-exposure. While Bettelheim found himself involuntarily in an extreme situation and Freud emphasized unconscious driving forces, a different perspective now comes to the fore: the conscious decision to expose oneself to extreme experiences—whether for spiritual, artistic, or existential reasons. This form of self-exposure points to a new dimension of the extreme: not as a fate that is suffered, but as a chosen path to transformation. In this context, Freud’s notion of the “oceanic feeling”—a sense of boundless unity described in *Civilization and Its Discontents* (1930)—offers a further example of an extreme inner state. While Freud interpreted it as a regressive return to early ego-boundlessness, it can also be seen as a liminal experience oscillating between narcissistic dissolution and spiritual transcendence. Such states illustrate how extreme experiences may arise not only from external events but also from symbolic inner processes.

This shifts the focus from reacting to the extreme to intentionally seeking out boundary experiences—a transition that is central to understanding the dispositional component.

## The Dispositional Component: Personal Need for Experiencing Extremes

Having discussed the situational component of extreme experiences in the sense of a sudden confrontation with existential borderline situations (Bettelheim, 1943, 1982) in the previous section, a further question now arises: How can extremes be understood if we look not only at external circumstances but also at internal dispositions that motivate people to expose themselves to such experiences? It is no longer just a matter of “passively enduring” extreme situations, but of actively seeking out, living through, and interpreting such experiences—and thus of what I refer to as the *dispositional component*.

To deepen this shift in perspective, it is necessary to consider how psychoanalytic theory itself has been expanded to account for cultural embeddedness. Examining a specific case study first requires a theoretical positioning of psychoanalytic concepts in a cultural context. Ethnopsychoanalysis, founded by Paul Parin, Fritz Morgenthaler, and Goldy Parin-Matthèy, combines classical psychoanalytic theory with cross-cultural field research. It assumes that unconscious processes do not arise in isolation within the individual, but are embedded in symbolic orders, linguistic practices, and social structures of meaning. In this context, Parin and colleagues emphasize that the unconscious does not operate in a culture-free manner, but is structured by the respective social conditions (Parin et al., 1980). This perspective makes it possible to avoid hastily classifying extreme experiences—especially those that manifest themselves in ritual practices—as pathological deviations, but rather to understand them as culturally framed expressions of inner conflicts, psychological transformations, and identity-related negotiation processes. This allows a conceptual bridge to be built between the theoretical considerations of Freud and Bettelheim and the lived experiences of people whose extreme states are located within specific cultural frames of interpretation.

A particularly revealing example is the case of Sirima documented by Obeyesekere (1981). Her story shows that extreme experiences are not necessarily pathological, but can also be understood as an expression of a profound inner struggle with existential questions. Sirima uses ritual practices such as fire walking not as an escape from reality, but as a symbolic act of self-empowerment and reorientation. Her experiences mark transitions—from family powerlessness to spiritual self-determination, from social exclusion to religious recognition.

The following case was documented by Obeyesekere (1981) as part of his ethnographic research on religious ecstasy and healing in Sri Lanka. He describes the spiritual development and personal challenges of a woman named Sirima, whose religious practices are closely linked to the pilgrimage site of Kataragama.

Sirima traveled to Kataragama for the first time when her second child was eighteen months old. The entire family took part in the pilgrimage, during which the child was weaned with rice in a ritual act at the shrine of Ganesha, adjacent to the main shrine of Skanda. On this occasion, she danced the Kavadi with her husband and experienced the fire-walking ceremony for the first time. She felt an inner urge to walk over the fire herself—a wish she fulfilled on her second visit to Kataragama.

This second trip took place two years after her father’s death, at a time when conflicts with her husband were becoming increasingly intense. Obeyesekere recounts: *She went alone*,* determined to walk the fire. She had heard that the divisional revenue officer of the area*,* a top government bureaucrat*,* was a strong believer in Kataragama and went to see him. This bureaucrat introduced her to Tennakon Maniyo (Mother Tennakon)*,* a venerable and respected fire walker who helped Sirima cross the fire. Since then she regards Tennakon Maniyo as her mentor. “I didn’t get burned*,* and so I felt a desire for me*,* I thought as I crossed the fire that the god-lord will help me overcome the problem at home.” But did you have problems at that time? “Yes*,* little by little… only it became much worse later.” Regarding that first experience with fire*,* she said*,* “I was happy since the fire was cool. I felt a sense of triumph since I was unscathed.” Why? “Perhaps because of the god’s love for me… maybe because of the god’s protective look over me. Since then I have walked over the fire thirty-six times at various pilgrimage centers. By fire walking my bhakti for the god increased*,* I can have peace inside even though I have troubles at home. I experience pleasure*,* vinoda*,* here; my mind is calm*,* I feel no pain.”*

As reported by Obeyesekere, Sirima’s family problems worsened as her religious devotion increased. Her husband reacted with verbal attacks on her pilgrimages to Kataragama and made derogatory remarks about the deity itself—apparently referring to its mythological relationship with a lover. He described Skanda as the god of the despised Tamil Hindus. When Sirima lit oil lamps for the deity at home, he threw them away and berated her, saying she was married to the god. Although the couple continued to live together, the situation became increasingly unbearable for Sirima. After the birth of her fourth child, her mother finally advised her to separate. Sirima then left the family home with her four children and moved in with her mother.

Despite everything, she continued to hope for reconciliation. In March 1971, she visited her mentor Tennakon Maniyo at her shrine in Maho (Northwest Province) to find out whether her family problems might be due to magic or witchcraft. Shortly thereafter, the uprising broke out in Sri Lanka in April 1971, trapping Sirima in Tennakon Maniyo’s house for 18 days. Upon her return to Buttala, she learned that her husband had entered into a new relationship with a seventeen-year-old girl from the neighborhood. This meant the definitive end of the marriage, which was soon formalized.

Sirima now lived independently in a house near her mother’s. She placed two of her children in the care of relatives in the Western Province. With remarkable initiative, she cultivated her small piece of land, organized pilgrimages, and traded rice, which she transported from her region to areas where it was scarce and sold it at a profit. She always appeared well-groomed and neat and could easily be mistaken for an educated middle-class woman.

However, her religious activities deviated in some respects from conventional Buddhist practices. As Obeyesekere (1981) describes, in 1972 she collected donations for the construction of a stupa in the local Buddhist temple and organized a three-day religious festival (pinkama). However, some of the rituals she initiated were explicitly intended to “attract crowds.” For example, she organized a fire walk on the temple grounds in which thirty people participated. Under the guidance of Mohotti Sami, a Sinhalese priest and entrepreneur from Kataragama, she had herself hung on hooks—a ritual that had previously been practiced exclusively by Tamil Hindus. Neither fire walking nor hook hanging were traditionally part of Buddhist temple rituals; rather, they were considered unorthodox and un-Buddhist. In Sirima’s village, this was the first time such practices had been performed in a Buddhist context. Like many other female ecstatics, Sirima was a religious innovator who introduced Hindu elements into conservative Buddhist culture. However, these innovations would hardly have been possible without the approval of Buddhist monks and laypeople, who were themselves influenced by the Hinduism of Kataragama.

Obeyesekere vividly describes how Sirima, through a series of intense religious experiences—in particular fire walking—did not slip into a mental crisis, but rather found a new form of self-empowerment and spiritual orientation. Her experiences are not an expression of mental disorder, but rather part of a subjectively meaningful process of transformation. Sirima’s fire walk is not only a religious ritual, but also a symbolic act of self-conquest and reorientation. Her statement *“I felt a sense of triumph since I was unscathed”* refers to a deep sense of self-efficacy that arises from the experience of remaining unharmed in the face of fire. This experience becomes a source of inner peace (*“my mind is calm*,* I feel no pain”*) and spiritual deepening (*“my bhakti for the god increased”*), even though she is simultaneously confronted with family conflicts and social exclusion.

As exemplified by the aforementioned example, I interpret such ecstatic practices not as symptoms of pathology, but as culturally embedded forms of *expression* of coping, searching for meaning, and identity formation. Sirima’s actions—firewalking, hook hanging, organizing religious festivals—are creative, performative responses to existential crises. She uses these “extreme” experiences to break free from a destructive relationship, gain social recognition, and establish a new role in her community.

From a cultural and religious-ethnological perspective, extreme experiences cannot be interpreted primarily as pathological phenomena, but rather as expressions of profound inner struggles with existential questions. They mark transitions, crises, and turning points in the lives of individuals, which can be symbolically processed and transformed through ritual and ecstatic practices. Rituals such as fire walking or hook hanging, as described in the case of Sirima (see Obeyesekere, 1978), open up spaces for symbolic repositioning. In these liminal states, individuals can discover new meanings, leave old identities behind, and develop new forms of self-positioning. The experience of “remaining unharmed” in the fire is not interpreted as an irrational act, but as spiritual confirmation and a sign of inner strength. The cultural context of such experiences is crucial: what might be classified as “psychotic” or “dissociative” in a Western medical context appears in a religious context as a calling, healing, or spiritual empowerment. The interpretation by the person affected is central to this. Sirima, for example, does not experience her actions as a loss of control, but as conscious, purposeful steps toward self-healing and overcoming family and social conflicts. This perspective calls for a reassessment of extreme experiences: they should not be hastily classified as clinically abnormal or in need of treatment, but rather understood as potentially catalytic moments that can enable individual development, meaning-making, and social reorientation.

### The Problem of Discrimination or the Notion of the Experiential Component

However, Filipp and Aymanns (2009) write that the characteristics of those affected, which may prove to be precursors to the occurrence of critical life events, are also of particular interest. The focus is reversed: it is not only about the effects that critical life events have on those affected, but the occurrence of such events is itself a phenomenon that requires explanation. Critical life events are by no means randomly distributed across the population; they are more than just bad luck and can sometimes even be reconstructed as unintended side effects or long-term consequences of human actions. External circumstances often play a role in the life events a person is confronted with: you cannot lose your job if you are retired; you cannot experience a breakup if you are not in a relationship; and you don’t have to deal with a child’s serious illness if you don’t have children. As trivial as these examples may seem, they point to the importance of biographical context and current life circumstances for the likelihood of events occurring. Of course, there are many life events that are beyond the control of those affected and prove to be completely independent of their actions and circumstances, which is not to be doubted in any way. These are the fateful experiences and events that often come to mind when we talk about critical life events. But we must look beyond them.

Particularly striking examples of this are extreme experiences embodied in the figure of the martyr: People who sacrifice their lives for a higher truth, moral conviction, or spiritual calling. Such figures, for example Mahatma Gandhi, Martin Luther King Jr., or Mother Teresa, are very unlikely to be remembered as pathological in collective memory cultures, but rather as heroic. Their extreme experiences, whether through persecution, self-sacrifice, or radical life choices they have made themselves, are interpreted as meaningful not only individually but also in a social context. This cultural reception shows in a special way that extreme experiences do not necessarily have to be accompanied by disintegration or loss of control, but can also be an expression of deep inner coherence and purposefulness. The decision to place oneself in the service of a greater cause—whether through nonviolent resistance, charitable work under extreme conditions, or spiritual asceticism—points to a form of self-transcendence that is considered the highest form of human development in many religious and ethical traditions.

The life and work of Mother Teresa thus become a paradigmatic example of a dispositional search for the extreme: her conscious decision to live and work in the slums of Calcutta among the poorest of the poor was not an expression of a pathological withdrawal from the world, but of a radically lived ethic of charity. She understood her experiences, marked by physical deprivation, spiritual darkness, and existential devotion, as part of a higher meaning. In this sense, the extreme is not only suffered, but actively chosen to serve as a path of transformation, self-realization, and social impact. Such an example makes it clear that extreme experiences are not only individually biographical, but also culturally and symbolically charged. They function as narrative anchor points of collective identity and moral orientation. The figure of the “positive martyr” thus exemplifies the transformative potential of the extreme and beyond pathologization, is to be understood as an expression of an existential decision for the good.

## Towards a Theory of Extreme Psychology

From a broader theoretical perspective within psychotherapy science and psychology, extreme psychology can be seen as a field concerned with the limits and transformative potentials of human experience: Extremes represent both the limits and possibilities of psychological life. They reveal our deepest vulnerabilities and our highest aspirations. This paper has traced how psychology has conceptualized extremes—as deviations, dangers, and, increasingly, as developmental or existential opportunities. I therefore make a conceptual distinction between two complementary but differently focused terms: *extremes in psychology* and *the psychology of extremes*.

The term *extremes in psychology* refers to the diversity of extreme phenomena as observed, described, and analyzed in psychological research and practice. It encompasses a broad spectrum of manifestations—from extreme emotional states and unusual behaviors to borderline experiences in religious, traumatic, or performative contexts. This perspective is comparative and open to contradictions: it allows different forms of the extreme to be juxtaposed and their similarities and differences to be identified. Examples include ecstatic self-transcendence in religious rituals (as in Sirima), loss of control in traumatic situations (as in Bettelheim), or extreme athletic or artistic performances. Psychology serves here as a lens through which these phenomena are viewed and systematically classified.

In contrast, the term *psychology of the extreme* aims at a theoretical consolidation: it refers to the attempt to understand the psychological mechanisms, structures, and dynamics that underlie experience and behavior in extreme situations. The focus here is no longer just on the phenomena themselves, but on the question of *what* makes an experience *extreme* in the first place—and *why*. It is about analyzing the psychological processes that are activated in moments of maximum intensity, shift in control, novelty, or identification and learn about there practical implications for treatment likewise psychotherapy. The dimensions developed, form the theoretical foundation for such a psychology of extremes. The goal is to present a consistent model that takes into account individual, cultural, and contextual factors.

In summary, while *extremes in psychology* describes the diversity of extreme phenomena within psychological contexts, *the psychology of extremes* aims to develop a theoretical framework that makes the extreme itself comprehensible as a psychological phenomenon per se. In the long term, this could give rise to an independent branch of research—a *psychology of extremes* that systematically examines the limits of human experience and behavior.

## Discussion

Each new moment in our lives can lead to an extreme situation. I argue that extremes in psychotherapy science and clinical psychology should not be viewed as inherently pathological, but rather as part of a broad natural spectrum of human experience. Human psychological functions are built on experience, which includes both positive and negative extremes. Extremes do involve risks and require increased caution but at same time can serve as catalysts for growth, insight, or cultural and individual expression. A crucial aspect of extreme experiences is their temporal structure. A crucial aspect of extreme experiences is their temporal structure. As Valsiner (2014) emphasizes, psychological phenomena are fundamentally unique and unrepeatable, as they emerge within the context of irreversible time: “The *same* thought or feeling—in a similar context—cannot occur again. A *similar* one can, but that means despite such similarity all the phenomena are unique.” (p. 7). Extreme experiences often unfold their meaning retrospectively and are marked by their singularity. This irreversibility turns them into narrative turning points that reshape not only experience but also the structure of the self.

Clinically, this clearly points to the need for integrative approaches that do not automatically suppress intensity, but rather help individuals make sense of their experiences. Furthermore, extremes cannot be represented on a sole scale of intensity, not sufficiently being aware how the notion of intensity is composed. Theoretical models need to be further developed to reflect the nonlinearity and transformation in human development, rather than being limited to stability and regulation. Culturally, it is crucial to question how psychological norms, whose construction can only follow the processing of extremes, work, especially in intercultural contexts. Definitions of “extreme” should not be treated as universal truths, but as historically conditioned judgments, which means they must be relativized and contextualized.

## Organizing Phenomenological Descriptions of the Extreme: A Six-Dimensional Framework

The construction of a psychology of extremes faces not only substantive challenges, but also fundamental methodological ones. As Meehl (1967, 1978, 1990) already pointed out in his critique of “soft psychology,” much psychological research suffers from a weak link between theory and empirical evidence, epistemic vagueness, and an excess of statistical formalization coupled with a lack of theoretical precision. The result is a research practice in which complex phenomena—such as extreme experiences—are either operationalized in an overly simplistic manner or prematurely pathologized.

In the sense of scientific model building (see therefore Suppes, 1993; Hempel, 1966; Lakatos, 1970), a model is not merely a descriptive structure, but a heuristic tool for generating, testing, and further developing theories. It acts as a mediator between abstract theory and empirical observation and must ensure both conceptual clarity and empirical connectivity. In the psychology of the extreme, this means that a model must capture the multidimensionality and context dependence of extreme experiences without reducing them to one-dimensional scales or categorical diagnoses.

The six-dimensional model presented below is designed as such a theoretical framework. It is based on the assumption that extreme experiences cannot be defined by a single characteristic, but rather by an interplay of structural dimensions, each of which relates to different aspects of psychological processing. The selection and formulation of these dimensions follows *the principle of a progressive research program* (Lakatos), that is, their capacity to generate novel hypotheses, explain existing phenomena, and foster interdisciplinary integration.

At the same time, the model is intended as a contribution to *pragmatic theory formation* (see Cartwright, 1983; Giere, 1999), which does not aim at universal laws, but rather at context-sensitive understanding and functional differentiation. It allows for flexible application in clinical, cultural-psychological, developmental-psychological, and social-scientific contexts and can serve as a basis for qualitative and quantitative research designs.

### Dimension 1: Intensity—Between Overwhelm and Numbness

#### Definition: Intensity Describes the Strength of an Emotional, Cognitive, or Physical Reaction

The dimension of intensity forms the energetic foundation of extreme experiences. It describes the strength with which emotional, cognitive, or physical reactions are experienced and is therefore a central criterion for the subjective assessment of a situation as *extreme*. In psychological literature, intensity is often understood as the quantitative value of a stimulus or sensation, represented on a continuum reflecting the strength of a behavior, such as an impulse or emotion (American Psychological Association, 2018). In this model, intensity is understood not only as a quantitative variable, but as a qualitative dimension that structures and shapes experience and frames its significance. Intensity also plays a central role in OPD-3 (Working Group on Operationalized Psychodynamic Diagnostics, 2023), particularly in the context of conflict and structural axes. Here, intensity is understood as a continuum that runs between functional and dysfunctional manifestations. On the conflict axis, for example, it describes how strongly an inner conflict influences experience and behavior—from subtle tension to overwhelming inner turmoil. On the structural axis, intensity manifests itself in the ability to modulate affect: excessive or insufficient affect intensity can indicate structural impairments. The OPD thus offers a differentiated diagnostic tool for understanding intensity not only as a symptom, but as an expression of psychodynamic processes.

#### Overwhelm—the Upper End of the Spectrum

At the upper end of the intensity scale is the experience of being overwhelmed. These are states in which emotional, cognitive, or physical arousal is so strong that it overwhelms the usual processing mechanisms. This form of high intensity can occur both in negatively connoted contexts (e.g., trauma, loss, violence) and in positively charged situations (e.g., religious ecstasy, birth, artistic inspiration).

In clinical practice, overwhelming experiences often manifest themselves in the form of flashbacks, panic attacks, or dissociative states. In EMDR therapy, for example, clients report “emotional waves” that they find difficult to regulate. These states are not only an expression of intense inner experience, but also an indication of a temporary disintegration of the self—a phenomenon described in psychodynamic theory as regression or ego disintegration.

Overwhelm is also a central element in cultural and ritual contexts. Sirima’s religious ecstasy, which puts her into trance states, is an example of a culturally framed form of high intensity. In such cases, the extreme experience is not pathologized but interpreted as spiritual opening or divine touch. This cultural framing significantly influences how intensity is experienced, interpreted, and integrated.

#### Numbness—the Lower End of the Spectrum

At the opposite end of the spectrum is numbness—a state of emotional, cognitive or physical under-arousal. While overwhelm is characterized by too much stimulation, numbness describes too little: the absence of resonance, meaning or inner involvement. In clinical psychology, this state is often associated with depression, dissociation or burnout.

A patient describes their life as “gray,” “empty,” or “like being behind glass”—even events that were once meaningful no longer elicit any reaction. In ritualized extreme situations, such as military training or nursing work under constant stress, repetition can lead to emotional numbness. Numbness can also occur in spiritual contexts—for example, when rituals are repeated mechanically without any inner involvement being felt. This form of underwhelming is no less extreme than overwhelming—it points to an existential emptiness that can be just as difficult to bear.

#### Transitions and Ambivalence

There are fluid transitions between overwhelm and numbness. In trauma therapy, for example, a shift between hyperarousal (overwhelm) and hypoarousal (numbness) can be observed—a phenomenon described as “oscillation” or “dissociative oscillation”. In spiritual practices, too, a phase of intense ecstasy can be followed by a phase of inner emptiness—a cyclical pattern described in many religious traditions.

These transitions show that intensity is not static, but dynamic. It depends on inner dispositions, external contexts, and cultural patterns of interpretation. An experience that is overwhelming for one person may seem empty or meaningless to another, depending on their biographical background, psychological structure, and social context.

#### Implications for Research and Practice

For research in relevant disciplines, the dimension of intensity means that extreme experiences cannot be captured solely by objective criteria (e.g., event intensity, physiological markers). Rather, subjective, narrative, and context-sensitive approaches are needed to understand the experience of intensity. Qualitative methods, diary analyses, or biographical interviews seem particularly suitable here.

In psychotherapy practice and clinical psychology, it is crucial to recognize the individual threshold for overwhelm and numbness. Therapists must learn to interpret subtle differences in intensity perception and adapt interventions accordingly. While some clients need to learn to protect themselves from overwhelm, others need support in coming out of numbness and reconnecting with themselves.

### Dimension 2: Impact—Between Transformation and Stabilization

#### Definition: Describes the Potential for Change Effect that an Experience has on Identity, Behavior, and Lifestyle

The dimension of impact describes the potential of an experience to bring about changes in a person’s thinking, feeling, behavior, or lifestyle. It is closely linked to the concept of biographical turning points and refers to the extent to which an experience influences an individual’s self-image, identity, or life structure—whether through upheaval, reorientation, or consolidation. In the theory of extreme experiences, impact is not only understood retrospectively (What has changed?), but also prospectively: What opportunities for development does an extreme experience open up or close off?

#### Transformation—the Potential for Profound Change

At one end of the spectrum is transformation—the ability of an experience to destabilize existing structures and enable new ones. Transformation here does not merely mean change in the sense of adaptation, but a qualitative restructuring of one’s relationship to oneself and the world. It can take place on various levels: cognitive (e.g., new beliefs), emotional (e.g., changed attachment patterns), existential (e.g., new sense of meaning), or social (e.g., role changes).

A classic example is the near-death experience, which leads to a radical reorientation in many of those affected: People quit their jobs, end destructive relationships, or begin a spiritual practice. In psychotherapy, too, a so-called “breakthrough moment”—such as recognizing an unconscious pattern or emotionally working through a repressed trauma—can lead to lasting change. In these cases, the extreme experience acts as a catalyst: it shatters the previous self-image and opens up new possibilities.

Sirima’s story is a paradigmatic example of such a transformation. Her religious practice not only changes her role within her family, but also establishes her as a spiritual leader in her community. The extreme experience—in this case, fire walking—is not experienced as an exceptional event, but as a turning point that establishes a new identity. Transformation here is not only individual, but also socially and culturally effective.

#### Stabilization—the Potential for Consolidation and Integration

At the other end of the spectrum is stabilization—the ability of an experience to maintain, consolidate, or restore existing structures. Stabilization is not the opposite of development, but rather its prerequisite: it creates security, orientation, and coherence in situations that could potentially have a disintegrating effect.

In extreme situations—such as in crisis regions, in the case of chronic illness or during existential transitions—rituals, routines or symbolic actions can take on a stabilizing function. A soldier in a war zone, for example, maintains his mental stability through daily rituals such as writing, praying or adhering to fixed routines. In addiction therapy, structured daily schedules help prevent relapses and regain a sense of control.

Sirima’s organization of religious festivals can also be understood as a stabilizing practice. It not only creates social recognition, but also a repeatable structure that gives her stability. In this sense, stabilization does not mean stagnation, but rather the ability to maintain psychological coherence in the midst of change.

#### Transitions and Interactions

Transformation and stabilization are not opposites, but complementary poles of a dynamic continuum. In many cases, a period of stabilization precedes a profound transformation—or vice versa: a transformation can only be sustainable if it is embedded in stabilizing structures. In psychotherapy, for example, it is necessary to first create a stable therapeutic setting before transformative processes can be initiated.

In addition, the same experience can have different effects on different people: what is a transformative crisis for one person may be a stabilizing confirmation of their values and way of life for another. The impact of extreme experiences is therefore not rooted in the event itself, but in the subjective processing and social context.

#### Implications for Research and Practice

For research in relevant disciplines, the dimension of impact means that extreme experiences must be understood not only as events, but as developmental junctures. Longitudinal studies are needed that capture not only short-term reactions, but also long-term changes.

In psychotherapy practice and clinical psychology, it is crucial to recognize the functional ambivalence of extreme experiences: they can have both disintegrating and integrating effects—often simultaneously. Therapists are challenged not only to regulate symptoms, but also to recognize and promote the transformative potential of extreme experiences. This requires an attitude that is not only focused on normalization, but also on understanding meaning and development.

### Dimension 3: Demand—Between Exertion and Ease

#### Definition: Refers to the Physical and/or Mental Effort (Workload) Required by a Situation

The dimension of demand describes the extent of physical, psychological, or cognitive strain that a particular situation or experience entails. It is closely related to the concept of “human workload” (Hart & Staveland, 1988) and to cognitive load theory (Sweller, 1988), which was originally developed in the field of learning but can also be used to understand extreme experiences. In this model, demand is not only understood as an objective quantity, but as a subjectively experienced burden that depends heavily on individual resources, situational contexts, and cultural patterns of interpretation.

#### Exertion—the Upper End of the Spectrum

At the upper end of the scale is effort—i.e., experiencing a situation as demanding, overwhelming, or exhausting. Such experiences are often characterized by a high density of stimuli, decisions, or physical exertion. They require the individual to mobilize inner resources that go beyond the everyday.

A classic example is work in highly stressful medical contexts: a nurse in an intensive care unit has to make complex decisions under time pressure, deal with emotional crises and be physically present at the same time. A mountaineer on Mount Everest is also exposed to extreme physical demands and mental exhaustion—here, physical limits, oxygen deprivation and existential threats all accumulate.

A particularly striking example of voluntarily sought-out exertion is Sirima’s hook ritual. The conscious decision to expose oneself to physical pain is not experienced here as destructive, but as spiritually meaningful. The effort is not only endured, but actively sought out—as a means of purification, self-conquest, and transformation. This points to a central insight: effort is not negative per se, but can—depending on the context and interpretation—be experienced as meaningful, identity-forming, or even healing.

#### Ease—the Lower End of the Spectrum

At the other end of the scale is lightness—the experience of a situation as effortless, fluid, or natural. Lightness does not mean that there are no demands, but rather that these are experienced as easily manageable or even pleasantly challenging. In psychology, this phenomenon is closely linked to the concept of flow (Csikszentmihalyi, 1990): a state of complete immersion in which high demands are met with high competence.

A musician who is completely absorbed in their playing during a concert, or a climber who loses track of time on a difficult route, experience high demands—but not as a burden, rather as a fulfilling challenge. Lightness can also arise in meditation: an experienced practitioner can sit still for hours without feeling any physical or mental effort. Sirima’s statement that firewalking was “cool” also refers to a subjective lightness despite objective danger—an indication that the assessment of demands depends heavily on one’s inner attitude.

#### Transitions and Ambivalences

There are fluid transitions between effort and lightness. A situation that is initially experienced as overwhelming can, through practice, ritualization, or a change in meaning, become an experience of lightness. Conversely, an activity that is perceived as easy can suddenly be experienced as strenuous due to external changes (e.g., time pressure, emotional stress).

Particularly interesting are ambivalent experiences in which effort and lightness occur simultaneously. In psychotherapy, for example, working through a trauma can be experienced as both extremely demanding and liberating. In spiritual practices, consciously going through pain or exhaustion is often associated with a feeling of inner expansiveness or lightness. This ambivalence points to the need to understand demands not in a one-dimensional way, but as a dynamic interplay of stress, meaning, and coping.

#### Implications for Research and Practice

For research in relevant disciplines, the dimension of challenge means that extreme experiences cannot be captured solely by objective stress factors (e.g., duration, intensity, complexity). Instruments are needed that take into account the subjective assessment of challenge—for example, through qualitative interviews, diary analyses, or psychophysiological measurements in combination with self-reports.

In psychotherapy practice and clinical psychology, it is crucial to recognize the individual stress threshold and to tailor interventions accordingly. While some clients need to learn to accept demands again (e.g., after a depressive withdrawal phase), others need support in protecting themselves from excessive demands. Therapists are called upon to understand demands as an area for development—not only as a risk, but also as a resource. The decisive factor here is the fit between inner capacity and external challenges—a (un)balance that must be renegotiated again and again.

### Dimension 4: Control—Between Perceived Gain and Loss of Control

#### Definition: Subjective Perception of Influence or Powerlessness Over a Situation

The dimension of control describes the subjective perception of influence, controllability, or powerlessness in relation to a situation or an internal state. It is a central element of extreme experiences, as it is directly linked to questions of autonomy, self-efficacy, and psychological integrity. In psychological theory, control is closely related to concepts such as locus of control (Rotter, 1966), self-efficacy (Bandura, 1977), and coping (Lazarus & Folkman, 1984). In the context of extreme experiences, control is not understood as an objective ability, but as a subjective experience—that is, as the feeling of being able to influence one’s own experience and behavior or not.

#### Gain of Control—the Upper End of the Spectrum

At the upper end of the scale is the experience of self-efficacy and control. In such states, the individual feels capable of dealing with challenges, making decisions, and influencing the course of events. This feeling can have a stabilizing, structuring, and identity-strengthening effect—especially in extreme contexts where external uncertainty prevails.

In psychotherapy, promoting self-efficacy is a central goal. A patient with an anxiety disorder who learns to regulate panic attacks not only experiences a reduction in symptoms but also a strengthening of their self-image. Sirima also regains control over her life and spiritual identity through her religious rituals. The repetition of rituals, the symbolic meaning of the action, and social recognition work together to create a sense of influence and orientation.

In trauma therapy, regaining control over memories is a central turning point. The ability to consciously regulate traumatic content, name it, and place it in a narrative context is not only therapeutically effective but also identity-forming. In this context, control does not mean total control, but rather the feeling of not being completely at the mercy of others—a feeling that is essential for the psychological integration of extreme experiences.

#### Loss of Control—the Lower End of the Spectrum

At the other end of the scale is loss of control—a state in which the individual experiences themselves as powerless, controlled by others or overwhelmed. Loss of control is a central feature of many extreme experiences, especially in traumatic, psychotic or existential crises. It can manifest itself as a feeling of powerlessness, disorientation or alienation from one’s own self.

A striking example is Bettelheim’s description of his arrest by the Nazi regime. The sudden break with all previous life structures, the complete surrender to a repressive system, and the experience that familiar values and strategies no longer apply mark a radical loss of control. In such moments, the ego is not only threatened, but its very foundation is shaken.

In psychotic episodes, too, the feeling can arise that thoughts or actions are being controlled from outside—a phenomenon described in clinical psychology as ego disturbance. The loss of control over one’s own thoughts, feelings, or actions is not just a symptom, but an existential experience that deeply affects the structure of the self.

#### Transitions and Ambivalence

There are complex transitions between control and loss of control. In many extreme experiences, phases of self-efficacy alternate with moments of powerlessness. In trauma processing, for example, reliving an event may initially appear as a loss of control, but with therapeutic support, it can be transformed into a feeling of control. In spiritual practices, too, temporary loss of control—for example, in trance states—does not necessarily have negative connotations, but can be understood as an opening to transcendent experiences.

Particularly interesting are ambivalent states in which control and loss of control are experienced simultaneously. An example is voluntary submission to a ritual that involves pain or a loss of boundaries: the action is consciously chosen (control), but the state that results is not completely controllable (loss of control). This ambivalence points to the need to understand control not as a binary category, but as well as a dynamic continuum.

#### Implications for Research and Practice

For research in relevant disciplines, the dimension of control means that extreme experiences cannot be understood solely in terms of external conditions (e.g., violence, illness, loss), but rather through the subjective experience of influence and powerlessness. Methods are needed to measure the quality of experience of control.

In psychotherapy practice and clinical psychology, it is crucial to understand the individual control structure and to strengthen it in a targeted manner. Therapists must recognize when clients relinquish control, when they regain it, and how they deal with loss of control. Especially when working with traumatized people, restoring control is a central therapeutic goal—not only on the behavioral level, but also on the symbolic, emotional, and narrative levels.

### Dimension 5: Novelty—Between Newness and Ritualism

#### Definition: Degree of Unfamiliarity with a Situation in an Individual or Cultural Context

The dimension of novelty describes the degree of unfamiliarity that an experience has for an individual or a social group. It refers both to the subjective perception and the cultural embeddedness of a situation and plays a central role in the evaluation of experiences as *extreme*. Novelty is not only a cognitive characteristic, but also an emotionally charged experience that can be accompanied by uncertainty, amazement, irritation, or fascination. In psychological research, it is closely linked to concepts such as novelty seeking (Zuckerman, 1994), ambiguity tolerance, liminal states (Turner, 1969), and cultural strangeness.

#### Newness—the Upper End of the Spectrum

At the upper end of the scale is newness—the experience of a situation as entirely new, unfamiliar, or unpredictable. Such experiences break with previous patterns of interpretation, challenge existing identity structures, and often create a feeling of disorientation or overwhelm. At the same time, they can also be experienced as fascinating, expansive, or transformative—depending on the context and inner disposition.

A classic example is the psychedelic experience: substances such as psilocybin or LSD can lead to radically altered perceptions of time, space, body, and self. For many people, these experiences are not only new, but beyond what they could previously imagine—they break the boundaries of everyday consciousness and open up new realms of experience. Sirima’s introduction of Hindu rituals into a Buddhist context also represents a form of cultural novelty that both creates social tensions and opens up new spiritual spaces.

Another example can be seen in migration: entering a new cultural environment can be an extreme experience because familiar norms, values, and forms of communication no longer apply. The new environment seems foreign, incomprehensible, or even threatening—a state described as cultural shock. At the same time, this unfamiliarity can also be experienced as an opportunity for reorientation and identity development.

#### Ritualism—the Lower End of the Spectrum

At the other end of the scale is ritualization—the experience of a situation as familiar, predictable, and structured. Such experiences are characterized by repetition, cultural embeddedness, and emotional security. They provide orientation, reduce uncertainty, and enable psychological stability—especially in extreme or crisis situations.

Rituals are a central means of establishing familiarity. Daily routines such as prayers, meals, or greetings structure everyday life and create a sense of continuity. In clinical contexts, structured routines—such as visits, medication administration, or therapy plans—convey a sense of security and predictability. Sirima’s repeated fire walks also show how an originally extreme experience becomes familiar through repetition. The action does not lose its meaning, but gains depth and integration through its repeatability.

Ritualized familiarity is not synonymous with boredom or stagnation. Rather, it provides a secure framework within which even intense or challenging experiences can be processed. In psychotherapy, for example, the recurring setting (same time, same place, same structure) creates a space in which new experiences can be made and integrated.

#### Transitions and Ambivalence

There are dynamic transitions between novelty and familiarity. An experience that is initially perceived as new and unsettling can become familiar through repetition, reflection, or cultural framing. Conversely, a familiar situation can suddenly appear new due to a change of context, a shift in perspective, or emotional reactivation.

Of particular interest are liminal states—transitional states in which old structures have been dissolved but new ones have not yet been established (see Turner, 1969; Zittoun, 2019). These threshold spaces are often characterized by a high degree of novelty, uncertainty, and ambivalence. They can be experienced as chaotic or creative, depending on whether new structures of meaning can be developed. Building on this, De Luca Picione and Valsiner (2017) describe liminal experiences as narrative transitional spaces structured by semiotic borders. These borders function as dynamic devices that connect past and future, self and other, and inner and outer realities. Liminality is thus not merely a transition, but a semiotically charged process of meaning-making, characterized by instability, ambiguity, and affective intensity. It is precisely within these intermediate spaces that novelty and creativity emerge, enabling the reorganization of identity and the formation of new narrative structures.

In such states, it becomes particularly clear that novelty is not just a property of the situation, but a relational phenomenon: it arises in the field of tension between experience, expectation and cultural interpretation.

#### Implications for Research and Practice

For research in relevant disciplines, the dimension of novelty means that extreme experiences must be understood not only in terms of their content, but also in terms of their experiential structure. Methods are needed that capture the subjective strangeness or familiarity of a situation. The analysis of rites of passage, initiations, or biographical breaks can also provide valuable insights.

In psychotherapy practice and clinical psychology, it is crucial to recognize the novelty quality of an experience and to provide therapeutic support. For some clients, it is necessary to expose themselves to new things again—for example, after traumatic paralysis or depressive withdrawal. For others, the challenge lies in integrating the new without being overwhelmed by it. Therapists are called upon to create a space in which novelty can be experienced not as a threat but as an opportunity—embedded in a network of familiarity, relationships, and meaning.

### Dimension 6: Identification

#### Definition: Degree of Personal Connection or Identification to an Experience or Situation

The dimension of identification describes the extent to which an individual feels personally connected to an experience, role, situation, or symbol. It concerns the question of whether and to what extent an experience is perceived as part of one’s own self—whether it is “mine,” whether it “affects me,” whether it “belongs to me.” Identification is a central mechanism of meaning-making and plays a crucial role in processing extreme experiences. In psychological theory, it is closely linked to concepts such as self-construction, narrative identity (McAdams, 1993), symbolic interaction (Mead, 1934), and emotional resonance.

#### Involvement—the Upper End of the Spectrum

At the upper end of the scale is deep involvement—experiencing something as deeply personal, meaningful, and relevant to one’s identity. In such states, emotional resonance is high, cognitive processing is intense, and social embeddedness is strong.

The experience is not merely “had”, but truly “lived”—becoming part of one’s personal history, self-concept, and worldview. An activist who identifies with a social movement experiences protests not only as political action, but as an expression of their innermost convictions. The experience is not external, but internally anchored—it becomes a stage for self-realization.

Sirima also sees herself as a spiritual servant of God—her rituals are an expression of her deepest identification and give her life structure, meaning, and direction. The extreme experience—such as fire walking or the hook ritual—is not experienced as an exceptional state, but as an expression of an inner truth.

In psychotherapy, recognizing one’s own patterns in the stories of others can lead to strong identification. Clients report that they “recognize themselves”, that they “feel that it applies to them”, or that “something inside them is touched.” This form of participation is highly effective therapeutically because it creates emotional depth, motivates change, and enables integration.

#### Detachment—the Lower End of the Spectrum

At the other end of the scale is detachment—experiencing something as external, observable, or not personally relevant. In such states, emotional involvement is low, cognitive processing is sober, and social embeddedness is weak. The experience is not experienced as part of the self, but as something that “happens to others”, that “one analyzes” or “professionally accompanies.”

An emergency doctor must remain emotionally detached in acute situations in order to be able to act. The ability not to identify with the suffering of patients is not indifference, but a form of professional self-regulation. In psychoanalysis, distance is understood as a prerequisite for reflection and analysis—it creates space for insight without severing the emotional connection. In research on extreme states, too, a certain distance is necessary in order not to be “drawn into” the world of those affected.

Distance should not be viewed negatively in this context. It can serve as a protective mechanism, a prerequisite for objectivity, or a means of self-preservation. In psychotherapy, for example, it is important that therapists do not “merge” with their clients, but maintain a reflective relationship that balances closeness and distance.

#### Transitions and Ambivalences

There are complex transitions between identification and distance. An experience that initially seems foreign or irrelevant can suddenly be experienced as deeply personal through biographical resonance, emotional activation, or social mirroring. Conversely, a formerly identity-forming experience can be viewed with distance through disappointment, trauma, or a change of perspective.

Particularly interesting are ambivalent states in which identification and distance occur simultaneously. In therapy, for example, a client may identify strongly with a topic but at the same time comment on it with ironic distance—a protective mechanism that allows closeness without causing hurt. In ritual contexts, too, an action can be experienced as both personally meaningful and culturally prescribed—a tension that can be productive or conflictual.

#### Implications for Research and Practice

For research in relevant disciplines, the dimension of identification means that extreme experiences must be understood not only in terms of their content, but also in terms of their subjective relevance. Methods are needed that capture personal meaning, emotional involvement, and narrative embedding.

In psychotherapy practice and clinical psychology, it is crucial to recognize the identification structure of an experience and use it therapeutically. For some clients, it is necessary to reidentify with themselves in the first place—for example, after traumatic dissociation or depressive alienation. For others, the challenge lies in breaking away from over-identified patterns—such as internalized feelings of guilt, rigid role models, or destructive self-attributions. Therapists are called upon to create a space in which identification is possible but not mandatory—a space in which closeness and distance are allowed to act as dynamic forces.

## Limitations and Future Directions

The six-dimensional model presented in this paper is conceptual and requires empirical validation. The dimensions were derived through theoretical synthesis rather than data-driven analysis, which limits the generalizability of the model across diverse populations and contexts. Furthermore, the model is grounded primarily in Western psychological and philosophical traditions. Although efforts were made to include transcultural understanding, further research is needed to explore how extreme experiences are conceptualized and processed in non-Western settings. Another limitation lies in the absence of clinical case studies or empirical illustrations beyond the example of Sirima. Future work should incorporate a broader range of examples, including clinical, artistic, and everyday contexts, to test the model’s applicability. Finally, while the model aims to be integrative and flexible, its practical implementation in therapeutic or research settings has not yet been tested. Future studies should examine how the six dimensions can be operationalized and applied in empirical research and clinical practice.

## General Conclusion: A New Perspective on the Human Condition

Extreme experiences are not a marginal phenomenon, but permeate the entire spectrum of human existence. We encounter them in everyday life, culture, science, and therapy—not as exceptions, but as expressions of our psychological depth. We actively create extremes: through our decisions, our values, our confrontations with borderline situations. They are not only everywhere—they are part of how we create meaning.

Ultimately, accepting the paradox of extremes allows psychology and psychotherapy science to gain a deeper understanding of human existence—in the recognition that, as Schiller wrote, we often attain wisdom only through madness. Madness, however, not in the English sense of the term “insane” and as a dichotomy to “sane,” but rather in its German usage, which understands it as a combination of ‘Wahn’ (delusion) and “Sinn” (meaning), but does not originally imply any pathological potential in the word itself.

Extremes are not pathological per se. They can be destructive, yes—but they can also be transformative, creative, and healing. Psychology must move away from the binary logic of “normal vs. disturbed” and understand extremes as potentially meaningful states that are relevant to development. What is considered a “disorder” today may be understood as a turning point tomorrow.

To do justice to this complex phenomenon, this article proposes an integrative model that describes extremes along **six dimensions**:

1. **Intensity**—between overwhelm and emotional numbness.
2. **Impact**—between transformation and stabilization.
3. **Demand**—between exertion and ease.
4. **Control**—between gaining control and losing control.
5. **Novelty**—between radical newness and ritualized familiarity.
6. **Identification**—between deep personal involvement and detached observation.

These dimensions enable a differentiated view of extreme experiences—beyond diagnoses, beyond norms. This also requires critical reflection on the concept of normality itself. In diagnostic systems, extreme experiences are often defined in contrast to an assumed norm—but this norm is rarely questioned in terms of its cultural, historical, or subjective construction. What is considered “normal” is not a neutral starting point, but a socially negotiated framework that can exclude, marginalize, or pathologize experiences that lie outside its boundaries. A psychology of the extreme therefore challenges the assumption that deviation is synonymous with disorder and invites us to understand normality as a dynamic, context-dependent construct rather than a fixed point of reference. They open up new perspectives for research, therapy, and social discourse. A psychology of extremes is therefore not just a theory about borderline experiences—it is a plea for the recognition of the diversity of human development. Future research should seek to refine and complicate the notion of extremes, resisting reductive binaries. Psychotherapy Science and Psychology must remain open to the idea that within what appears as excess, there may lie a path to meaning, connection, and even peace. A psychology of extremes thus opens up new perspectives not only on deviance, but also on human evolvement itself—in all its depth, contradictions, and capacity for change.

## Data Availability

No datasets were generated or analysed during the current study.

## Footnotes

1 German original: „Wir gelangen nur selten anders als durch Extreme zur Wahrheit – wir müssen den Irrtum – und oft den Unsinn – zuvor erschöpfen, ehe wir uns dem schönen Ziele der ruhigen Weisheit hinaufarbeiten.“ (Schiller, 1786/1962), p. 336).

2 The current version of OPD-3 has no official English translation. For English-language reference, please consult OPD-2, (2008).

3 The term “extreme” is used in this article exclusively in the sense of psychological experience at the limits of human capacity—for example, to describe intense emotional, cognitive, or existential states. Political or ideological forms of extremism—as discussed by Kruglanski and Moskalenko (2025) in their book *The Psychology of the Extreme*—are not the subject of this paper. Although the authors use the term *extreme psychology*, they refer to motivational one-sided states (e.g., fanaticism, ideological fixation), not to the phenomena of subjective intensity, control change, or existential transformation examined here. The theory of extremes developed here aims at a differentiated understanding of human experience—beyond political attributions and ideological contexts.

4 Robinson (1970) criticizes Bettelheim’s psychoanalytic interpretations of the Holocaust. He argues that Bettelheim’s interpretation of psychoanalytic concepts lacks historical and social context. He further argues that the application of psychoanalytic concepts therefore takes place in a kind of vacuum, without taking into account the specific historical and political realities of the Holocaust. Ultimately, this has led to inaccurate and potentially harmful interpretations in psychology. Des Pres (1979) offers further criticism, which he concludes as follows: “The problem is compounded by the fact that, whereas survivors take exception to Bettelheim’s assessment of their experience, the literary and scholarly community still largely accepts Bettelheim’s position as the final word on men and women caught in extreme situation.” (p. 620).

5 On November 7, 1938, Herschel Grynszpan, a Polish Jew, entered the German Embassy in Paris and shot Ernst vom Rath, the embassy’s third secretary. Von Rath died of his injuries two days later. This event was used by the Nazi authorities as a pretext for a coordinated campaign of violence (also known as Kristallnacht or the November pogrom) against the Jewish population in the German Reich, during which numerous people were killed and many more were sent to concentration camps [for details, see Cuenot (1982)]

6 In the *Standard Edition* of Freud’s works, the German term *Trieb* is translated as *instinct*. However, contemporary psychoanalytic scholarship widely prefers *drive* to reflect the concept’s psychodynamic and symbolic nature, distinguishing it from biologically fixed instincts (*Instinkt*). For this reason, I will use the term *drive* throughout this text when referring to Freud’s theory—except in direct quotations, where the original translation is retained for historical accuracy.

7 “In the early stages of his researches, man believed at first that his dwelling-place, the earth, was the stationary centre of the universe, with the sun, moon and planets circling round it. […] The central position of the earth, moreover, was a token to him of the dominating part played by it in the universe and appeared to fit in very well with his inclination to regard himself as lord of the world. […] The destruction of this narcissistic illusion is associated in our minds with the name and work of Copernicus in the sixteenth century. […] Even the great discovery of Copernicus, therefore, had already been made before him. When this discovery achieved general recognition, the self-love of mankind suffered its first blow, the cosmological one.” (p. 139).

8 “In the course of the development of civilization man acquired a dominating position over his fellow-creatures in the animal kingdom. Not content with this supremacy, however, he began to place a gulf between his nature and theirs. He denied the possession of reason to them, and to himself he attributed an immortal soul, and made claims to a divine descent which permitted him to break the bond of community between him and the animal kingdom.” (p. 139) […] We all know that little more than half a century ago the researches of Charles Darwin and his collaborators and fore-runners put an end to this presumption on the part of man. Man is not a being different from animals or superior to them; he himself is of animal descent, being more closely related to some species and more distantly to others. The acquisitions he has subsequently made have not succeeded in effacing the evidences, both in his physical structure and in his mental dispositions, of his parity with them. This was the second, the biological blow to human narcissism.” (p. 139–140).

9 The third blow, which is psychological in nature, is probably the most wounding. Although thus humbled in his external relations, man feels himself to be supreme within his own mind. Somewhere in the nucleus of his ego he has developed an organ of observation to keep a watch on his impulses and actions and see whether they harmonize with its demands. If they do not, they are ruthlessly inhibited and withdrawn. His internal perception, consciousness, gives the ego news of all the important occurrences in the mind’s working, and the will, directed by these reports, carries out what the ego orders and modifies anything that seeks to accomplish itself spontaneously. For this mind is not a simple thing; on the contrary, it is a hierarchy of superordinated and subordinated agencies, a labyrinth of impulses striving independently of one another towards action, corresponding with the multiplicity of instincts and of relations with the external world, many of which are antagonistic to one another and incompatible.” (p. 140).
